# Professor Thomas E. Bond

**Published:** 1872-12

**Authors:** 


### IN MEMORIAM.

At a special meeting of the Faculty of the Baltimore College of Dental Surgery, convened October 7th, 1872, there were present Professors Austen, Gorgas, Noel, Howard, DeRosset and Harris.

The Dean announced the death, since the last meeting, of PROFESSOR THOMAS E. BOND, senior member of the Faculty, who departed this life on the 19th day of August, 1872, in the fifty-ninth year of his age.

Whereupon the Faculty adopted the following expression of their great bereavement:

In the death of Professor Thomas E. Bond, the last surviving originator of the plan of Dental Collegiate Education, the profession at large has sustained an irreparable loss.

Students of the Baltimore College will long remember his rare power and fascination as a lecturer; whilst his colleagues must ever mourn their deprivation of his warm and cheering friendship, the benefit of his sagacious counsel, and the support and encouragement of one so widely known as an eminent christian scholar.

Great as is the loss, there is, however, yet a greater; and this faculty respectfully tender their deepest sympathy to those from whom the Heavenly Father has taken so tender and wise a husband, father and friend.

